# Correction to “Impedance‐based remote monitoring in patients with heart failure and concomitant chronic kidney disease”

**DOI:** 10.1002/ehf2.14723

**Published:** 2024-03-15

Wintrich J , Pavlicek V , Brachmann J , Bosch R , Butter C , Oswald H , Rybak K , Mahfoud F , Böhm M , Ukena C . Impedance‐based remote monitoring in patients with heart failure and concomitant chronic kidney disease. ESC Heart Fail 2023;10: 3011–3018.10.1002/ehf2.14387 37537796 PMC10567629

The below funding statement for this article was missing:

Open Access funding enabled and organized by Projekt DEAL.

